# Pathology and Biobanking

**DOI:** 10.5146/tjpath.2020.01482

**Published:** 2020-05-15

**Authors:** Canan Kelten Talu, Muhammed Hasan Toper, Yasemin Şahin, İbrahim Halil Erdoğdu

**Affiliations:** Department of Molecular Pathology, Graduate School of Health Sciences, Dokuz Eylul University, Izmir, Turkey

**Keywords:** Biobank, Human tissue biobank, Pathology-centered biobank, Quality control, Sustainability

## Abstract

Biobanks are units where high quality and long-term protection of biomaterials is maintained. This system, in which biological materials and data are systematically recorded and stored, is a unique resource for the study of the pathophysiology of disease, the development of diagnostic biomarkers, and working with human tissues for the potential discovery of targeted therapeutic agents. At this point, the pathology unit plays a unifying and complementary role between the clinical and core disciplines and offers optimal management of the patients’ biomaterials for diagnostic and research projects. The aim of this article is to present general information with regard to a biobank constructed for the storage of tumor tissue and blood biospecimens.

Ethical issues (informed consent, protection of confidentiality and privacy, and secondary use of biospecimens) and the information technology system (collection, systematic recording, backup and protection of clinical information) are important issues in biobanking. The selection of freezers to be used in storage (mechanical freezers, liquid-vapor nitrogen tanks), and if mechanical freezers are preferred the establishment of the relevant infrastructure and support team (such as additional power units for protection from power outages), the preservation of materials by aliquoting in different freezers, ensuring financing so as to afford the cost of the infrastructure, and implementation of all these dynamics while adhering to international guidelines are of the utmost importance.

## INTRODUCTION

It was during the mid-1990s that researchers began to consider collections of biospecimens as a resource for their projects (1). However, the value of biological materials reached the biggest impetus with sequencing of the human genome in 2001. Additional popularity of “biobanks” was ensured by a Time Magazine article in 2009 where biobanks were identified among the top 10 ideas changing the world with regard to health and well-being (1).

*A biobank* is a valuable resource to access high-quality human biospecimens while preserving them over the long term. Those biospecimens may be used to elucidate the pathophysiology, diagnoses, and finally the treatments of diseases (2). *Biobanks* (also called *Biorepositories) *are defined as the infrastructures that enable the collection, handling, storage, retrieval, and distribution of biospecimens (3). A high-quality biorepository should adhere to standard operating procedures (SOPs) and disseminate best practices for annotating, collecting, processing, storing and retrieving biospecimens (4). The term ‘*Biological Resource Centre* (BRC)’ describes the combination of infrastructure, facilities, and resources. Therefore, tumor biobanks are basically BRCs. The Organisation for Economic Cooperation and Development (OECD) describes biobanks as service providers and repositories of living cells, organisms, cells and tissues, and of information relating to these materials (5).

Biobanks comprise a wide range of specimen types and sample collection formats, ranging from population-based biobanking of specimens from healthy individuals to specific diseased tissue specimens obtained by surgical interventions (6). Thus the main objective of biobanks is to share their collections with national/international communities in order to improve biomedical research and health care (5,7). The protection of the privacy of sample donors and informed consent must be ensured for biobanks (8). Furthermore, bioinformatics, long term financial support and administrative sustainability are the other important issues for maintaining biobanks (9).

Pathology is the fundamental component of hospital-based tissue biobanking (9,10). Pathologists provide essential diagnostic information for the treatment of the patients and can also make decisions on the sampling of tissues for the biobank as well as optimal preservation of biospecimens. Tissue biobanking is of particular importance for the use of novel biomarkers in clinical trials and the application of new technologies. Pathologists therefore act as a bridge between the clinicians and the researchers. In this review, we aimed to present information related to tumor biobanking.

## ETHICAL AND LEGAL REGULATIONS FOR BIOBANKS

The developments in science and technology in the last half of the twentieth century and the vast accumulation of knowledge in health services, medical applications and biomedical sciences have resulted in new ethical problems where traditional medical ethics principles are inadequate. In the Medical Ethics Manual published by the World Medical Association, it is stated that bioethics is a more comprehensive field than medical ethics (11). The subject of bioethics, which has especially developed following the progress made in modern gene technologies, requires a global approach to ethics. Gene technologies have first aimed to add new perspectives to research, as well as contributing to a better quality of life for human, animal and plant species. However, they have been alarmingly found over time to potentially lead to abuse of technology and unpredictable damage.

In addition to the problems brought about by modern gene technologies, the concept of “value” has been questioned again in various conditions emerging in the field of bioethics (such as research using human subjects, artificial termination of pregnancy, prenatal diagnostic methods, genetic counseling, assisted reproductive techniques, definition of death, and organ transplantation) (12). Physicians and health professionals interested in this issue had to make health-related decisions in such an area where “values” were being questioned. The Universal Declaration on Bioethics and Human Rights was adopted at the UNESCO General Conference assembled on October 19, 2005 in order to propose principles related to these issues and the relevant decisions to be made, and an important contribution was made to the field of bioethics (13). The Declaration, although not binding, is an international legal document that comprehensively emphasizes the relationship between human rights and bioethics. This document sets out the following bioethical principles;

- Human Dignity and Human Rights
- Benefit and Loss
- Autonomy and Individual Responsibility
- Consent
- Consent of individuals who are not competent to give consent
- Respect for the individual and respect for the integrity of the individual
- Privacy and Confidentiality
- Equality, Justice, Equity
- Non-discrimination, non-stigmatization
- Respect for cultural differences and pluralism
- Solidarity and Cooperation
- Social responsibility and Health
- Common Benefits
- Protecting next generations
- Protecting the environment, biosphere and diversity of the species (13).

The main objective of the Universal Declaration of Bioethics and Human Rights is to bring together the fundamental principles for biomedical research and clinical practice in accordance with the international law on human rights (13). In this respect, it is an important step in the quest to develop bioethical standards worldwide. However, the declaration has no intention of developing a new bioethical principle.

Biobanking has become more popular with the completion of the Human Genome Project. The conditions that make biomarketing legally hazardous can be specifically listed as follows (14):

- Biospecimens can be easily obtained without the consent of the person concerned,
- Biospecimens can be easily misused,
- The genetic data obtained are permanent personal information,
- They contain very important data for the life of the person concerned,
- They contain information about the family and the group of the person concerned;
- The data carry the potential to be used for discrimination,
- The use of the data by others creates scientific and commercial opportunities,
- There is the possibility of neglecting personal rights in case of misuse.

The predominant ethical issues related to biobanks include informed consent, protection of confidentiality and privacy, the secondary use of biospecimens, and profit sharing.

### Informed Consent

Informed consent is an important concept in research as well as applications of therapeutic medicine. On the other hand, biobank-based research has led to the need to reconsider traditional research ethics. In particular, developments in research using human-based biological materials have necessitated the identification of a new informed consent model for biobank research (15). The informed consent obtained from the subjects for biobank research is more comprehensive and detailed than the ones used in daily practice. Consent for biobank research should therefore be obtained in written form and signed by the subject. Various provisions have been established by international institutions (such as the World Medical Association, European Union and UNESCO) for the realization of informed consent in daily life. The basis for this sensitivity goes back to the Nuremberg Code (1947) and the Declaration of Helsinki (1964), which prohibited the inhumane use of individuals (16). Although the principles of informed consent cannot protect the individual on their own, they allow making a decision on how the materials and data to be obtained in the research will be used (16)*. *In case of biological materials, it can be predicted that the data obtained as a result of the research may pose a risk to the participants (17).

The consent forms to be used in biobank-based studies generally include the following items (15,18,19);

- Respecting the autonomy of the participants, and recognizing the benefits and risks, if any,
- Type of biospecimens to be collected and the relevant data,
- Research projects, objectives and research data,
- The purpose of the research (the collection of samples for a particular study requires a separate consent system. In other words, the participants provide consent for a single study. Biobanking requires a different consent in order to provide a source where the long-term stored samples can be used in many studies),
- Storage period of the biospecimens,
- Principles of sharing the data and biospecimens with other research organizations,
- Principles and procedures for biospecimen and data access by the researchers,
- Obtaining permission to collect health-related records from other databases,
- Procedures for re-contacting the participants;
- Regulations on the privacy and confidentiality of the participants;
- Restrictions for anonymization procedures and re-identification of the biospecimens;
- Feedback for the research results and methods of feedback;
- The right to withdraw from the research;
- Arrangements in case of failure or death;
- Regulations regarding sharing of gains;
- Possible commercial agreements indicating that the participants will not earn any business profits.

Although the consent process is implemented by many countries, it may vary in terms of national regulations. This may cause problems in international projects that require the use of biobanks (15).

Biobank-based studies are divided into two groups as those related to retrospective biobanks (previously collected biospecimens) and prospective biobanks (newly collected biospecimens) according to the collection time of the biological material and data contained. Informed consent indicates different conditions for studies on these two groups of biobanks.

### Informed Consent in Retrospective Biobank Research

Informed consent for retrospective biobanks containing previously collected and archived biological materials in medical care units and their data is a controversial issue (15,20). The general trend in legal regulations is to permit the use of previously collected biological biospecimens and related data without an approval if they are anonymous and meet certain conditions (15,20). However, consent requirements may vary from country to country depending on the current legal situation. This also affects the evaluation period of the consent process.

### Informed Consent in Prospective Biobank Research

A prospective biobank is established to create new infrastructures at the national and international level so that biospecimens can be used in projects that are scheduled at the time of collection or later on. A large number of national and international participation for each project requires all parties to provide the same type of consent (if possible). The relevant regulations include some rules that ensure the autonomy of the participants such as volunteering by the participants, comprehensibility of any information to be obtained, and systematically providing such information before starting any research project (20).

The informed consent forms for biobank research should include the following concepts as identified by the international literature and documents on ethics (15);

***General consent:*** Consent allows the use of biological biospecimens and related data at the time of their acquisition and in the future.

***Specific informed consent*** **:** Permits the use of biological biospecimens and associated data only for the research project at the time they are received.

***Partially limited consent:*** Permits the use of biological biospecimens and associated data for current specific and potential future research.

***Multilayered consent*** **:** Requires a detailed description of the various options related to the research topic.

***Dynamic consent:*** This is an internet-based interactive consent model that has been developed in recent years where the participants play an active role in the decision-making process

### Confidentiality and Privacy

The personal information contained in biobanks is considered private information. The use of this information is regulated by the consent of the individual and the decisions of the researchers, ethics committees and government bodies. Protecting the identities of biobank data holders is important both to ensure the confidentiality of the participants and to protect the privacy of the biospecimens and related data (15).

There are four main sample types in biobanks as regards protecting the privacy of the participants. These are:

- **Anonymous biospecimen:** Biospecimens obtained during community screening from individuals with unknown identity
- **Anonymized biospecimen:** Biospecimens that were obtained from identified individuals but anonymized before being studied
- **Identifiable biospecimen:** Biospecimens belonging to known individuals whose data are used as encrypted and coded information.
- ** Identified biospecimen:** Biospecimens belonging to known individuals whose personal information is exposed (21).

The most commonly used method is *coding *or *anonymization*. The coding method allows reestablishing contact with the owner of the data. This is advantageous in terms of providing new data for prospective studies. However, it is open to the improper use of information by authorized persons and may not provide complete security for participants (22). Anonymization ensures elimination of the link between biospecimens and their associated data with the participants, either reversibly or irreversibly.

### Secondary Use of Samples and Profit Sharing

Other important ethical issues include commercialization, patenting of the collected biospecimens and related data, and who will and in what terms benefit from the biospecimens and research. Ethical and legal regulations that protect the rights of both donors and researchers are required for research using human biospecimens. Options to participate, not to participate, or withdraw from the trial should be offered to the participants (15).

### Legislation in Turkey

Turkish legislation has no regulation specific to biobanks yet. However, a legal infrastructure needs to be constructed in order to establish biobanks in Turkey and to make sure that they operate in accordance with international standards. The relevant law should be enacted within the framework of the European Union directives.

Although the Turkish Draft Law on a National DNA Database and National DNA Databank prepared by the Ministry of Justice on January 17, 2007 was submitted to the Prime Minister’s Office, the draft was returned for review on April 14, 2008. No other draft bill has been submitted since then (14,23,24). The draft justification states that “DNA is personal data that contains a lot of confidential information about the person, and it is very important to comply with the international conventions our country is a part of as a third party regarding the use and protection of this data, and to refrain from violating the personal rights defined in our Constitution”. The definitions of the biological sample, DNA, DNA Analysis, DNA Profile and DNA Database terms have been included in the second article of the draft bill. The basic criteria related to DNA data have been defined under the &quot;Basic Principles&quot; section of the draft.

Accordingly, article 4 introduces the following regulations related to DNA data (14):

- Processing of DNA data in accordance with the law and general principles of integrity,
- Collecting DNA data only for legitimate purposes as stated in the relevant articles of the law and refraining from using and transmitting the data otherwise,
- Ensuring that the DNA collection is relevant, sufficient and appropriate for the purpose,
- Keeping the data until the end of the period specified by law,
- Ensuring accuracy of the data and providing updates as required.

The draft also addresses the subject of providing information in the “Principles of Volunteering and the Obligation to Provide Information”. However, the relevant legislations, ethics committees and consent form have not been developed for biobanks in Turkey yet and the adaptation process will need to be realized quickly.

## INFORMATION TECHNOLOGY IN BIOBANKING

An information technology (IT) system is a very important part of quality management as the entire process for each material collected in the biobank can be recorded (7). First of all, it provides a rich and high-quality information pool including the clinical information and consent documentation related to solid tissue and liquid biomaterials. In addition, it makes the journey of the biomaterial easily traceable by recording relevant data (information related to collection, processing, preservation times, storage properties and localization of the material in the repository, ambient temperature) (25). Although most pathology reports provide systematic information on the cases, it is recommended to use standard terminology based on international nomenclature to optimize data in biobanking (26)

The biobank document contains clinical, pathological and other information of the subjects together with the following parameters (5):

- Gender and date of birth, patient identity, demographic data, and status of vital signs.
- Diagnostic data, clinical stage (cTNM)
- Sample information, sampling date, and details of sample quality, collection method, stabilization process and preservation.
- Lesion information; tumor information (primary or metastatic), histological type, size, pathological stage (pTNM), grade, and other important data specific to the disease state
- Type, number, size, characterization of sample data (for tissue sampling; tumor focus, area adjacent to the tumor, sampling away from the tumor), and preanalytical data.
- DNA or RNA data such as concentration, purity, integrity.
- Storage information; the type of storage used, the localization of the material in the storage area and the ambient temperature.

Information technology applications have been developed to address biobanking processes, optimize workflow efficiency, and provide quality assurance. This system can manage data obtained from molecular research and provide controlled access to data acquired from the research community. In this respect, biobank system software is designed on the basis of security and robustness (7). Numerous software programs are available to assist in the use of information technology in tumor biobanks (3). With these software programs, all operations and procedures, including information about the operator, the equipment used, reagents and other consumables, can be recorded with the time also specified (27). Similarly, the process of sample collection, sample acceptance forms, processing and anonymizing of samples can be performed electronically to reduce user-related errors due to manual entry of data, and to improve workflow efficiency and quality assurance (7). Most software programs allow tracking the position of biospecimens in the biobank storage units and the procedures they are subjected to over time. Depending on the total number of samples to be stored, these programs can therefore propose sample storage space for new material and optimize space utilization. The software programs enable recording of all kinds of information on the processing and storage of biomaterials. This recorded information should include not only the process flow, but also the non-conforming situations during the process, whether corrective measures have been taken, and at which stage if so (5).

Software programs can also be used to monitor and manage donation approval. For example, a copy of the informed consent can be saved to the system, allowing biospecimens to be used only with the donor’s consent (5). Software programs can also monitor the distribution of tissue samples registered in the system and all processes related to those biospecimens. For example, requests and the transfer and return processes related to the biospecimens can be recorded as of the date of their realization *Material transfer agreements* related to the subject can also be stored in the system. Furthermore, data obtained from the research performed with the use of biobank materials can be added to the biobank database, thus contributing to the continuous development of data. Taking the above issues into account, when deciding on the software system to be used for the biobank one should be careful regarding topics such as security, robustness, data to be recorded, data quality, recovery of data, and the possibility to backup stored data to alternative databases at regular intervals (5,28). In particular, it is very important to protect and anonymize the identity data of the donors as the donors should always be anonymized (3,5).

## COLLECTION AND PROCESSING OF BIOSPECIMENS

All the surgical excision specimens obtained from the different types of Surgery Departments are gathered and processed in the Pathology laboratory with the intention of diagnosing the disease. Therefore, pathology laboratories and the pathologists have a central role in tissue biobanking. A pathologist can evaluate the specimen macroscopically and then make appropriate sampling of the tissue for both diagnostic purposes and for biobanking concurrently. The medical and scientific expertise of the pathologist is important in terms of making diagnostic decisions as well as managing the procurement and preservation of the surgical excision materials. Thus, the pathologist has an essential role to provide continuity between medical care and research (10).

Specimens collected during clinical practice include tissues, fluid samples, and body secretions. Samples for diagnosis and research samples should be taken from the materials at the same stage if possible and collected in separate containers for biobanking. A biobank has to evaluate all the different uses of the samples in the future and maximize the conditions for the survival and potential use of these samples (29).

In molecular studies, frozen tissue (-80°C to -190°C) and formalin-fixed, paraffin-embedded (FFPE) tissues are known to have their own advantages and disadvantages (30,31). Although histological detail in frozen tissue is microscopically lower than in FFPE tissue, frozen biospecimens are ideal for DNA/RNA, genome amplification, genome sequencing and cDNA microarray analyses (32). Frozen tissue samples therefore often offer sufficient quality for molecular studies. In addition, proteins in frozen tissue specimens are protected from degradation including enzymatic activity, while protein loss is seen in their FFPE samples (33).

Accurate recording of preanalytical details is crucial for immunoassay, molecular or proteomic analyses of biospecimens containing tissues and fluids (29). Information on the parameters affecting the preanalytical processes regarding these materials will be given in more detail below.

### Tissue Samples

Human tissues can be obtained from surgical materials or autopsy samples. While delivering these tissues to the pathologist, it is essential to minimize the duration of warm ischemia (the time spent at room temperature until tissue is removed from the human body and treated with fixation material) and cold ischemia. If possible, the container containing the tissue samples should be delivered on wet ice cubes and stored in ice or at 4°C in the refrigerator until it is placed into fixing solution to limit changes at the cellular level (29). The ischemia process has been shown to affect the values of biomarkers detected using molecular techniques or standard immunohistochemistry. In one study, needle biopsy specimens of breast cancer and excision materials sent after surgery have revealed various PI3K pathway markers. It has been reported that this difference may be due to the loss of phosphorylation in the material during surgery or the process of cold ischemia (34).

For solid tissue samples to be fixed, it is also necessary to specify the type and duration of fixation and the duration and conditions of storage. Optimum fixation of biospecimens depends on variables such as the volume of the fixative, tissue ratio, fixation time, temperature, and tissue thickness (35). Formalin fixation of biospecimens leads to fragmentation of nucleic acids (36,37). Since the length of fixation also affects nucleic acid quality, a fixation time of 6-18 hours for biopsy specimens and 12-36 hours for surgical specimens is recommended. However, 70% ethanol or alcohol-based fixatives can yield higher quality nucleic acids compared to formalin fixation and can be more suitable for molecular studies (37). A commonly preferred tissue preservative, RNALater, has recently been shown to provide better quality RNA and gene expression profiles compared to snap-frozen samples or formalin-fixed paraffin-embedded (FFPE) samples when used on tissue samples (38,39).

### Liquid Biospecimens

Liquid biospecimens (e.g., whole blood, plasma, serum, urine, bronchoalveolar lavage, saliva, ascites, tear fluid and seminal fluid) include cells, proteins, lipids, and metabolites that can function as biomarkers. The preanalytical information required for liquid materials includes the type of primary collection tube, delay time and temperature before centrifugation, centrifugation conditions, as well as long-term storage time and temperature (29,40).

The container used to collect a blood sample is itself an important preanalytical variable. These tubes and their content may contain stabilizing agents for anticoagulants or nucleic acids and thus affect the analysis of biomarkers in the blood sample. The tube content has been shown to affect proteins, hormones and other biomarkers used in various analytical techniques (41,42). In addition, some studies investigating the waiting time before centrifugation have shown that this duration may affect protein measurements in blood samples. Ayache et al. reported significant changes in plasma protein levels within 2 hours (43) Similarly, Banks et al. reported the development of important changes in low-molecular weight serum proteins within 30-60 minutes (44). In another study, it was reported that proteins remain stable by centrifugation of samples within 2 hours and then freezing within 2 hours after centrifugation (45). The temperature at which the samples are kept is another factor affecting protein stability. Significant protein losses were observed when the samples were kept at room temperature for more than 4 hours or kept at 4°C for 24 hours (46).

Blood is one of the most widely used biomaterials and contains various fractions such as plasma, serum, white blood cells and red blood cells. Tube types compatible with the intended test(s) should be used for blood collection (47,48). A collection tube containing silica or thrombin-like coagulation accelerator is required for studies in which serum will be used. Anticoagulated blood (consisting of plasma, buffy coat, and RBCs) is preferred for DNA- or RNA-based analyses.

Although there are several types of anticoagulants, blood samples stabilized with citrate provide higher quality RNA and DNA yield compared to other anticoagulants (47). EDTA-coated collection tubes are suitable for various DNA- and also protein-based assays; however, these tubes are not sufficient for cytogenetic studies (47,49).

The collected blood samples can be divided into fractions. Serum and plasma can be used for the analysis of protein, lipid, small molecules, and nucleic acid (48). Cell concentrates can be used in functional studies and also in flow cytometry as a source for culture experiments or cellular nucleic acids (48,50). Since blood components can maintain their viability at room temperature for up to 48 hours, the time between collection and processing of the samples should be kept short (less than 24 hours if possible).

A variety of Standard Operating Procedures (SOPs) of the International Society for Biological and Environmental Repositories (ISBER), the National Cancer Institute (NCI), and other organizations exist and systematically describe the process of collecting and processing the tissue and fluid (blood) biospecimens described above (40,51).

The liquid biopsy procedure is an important development that has recently been introduced for the molecular profiling of cancer patients. Various biomarkers reflecting tumor-specific changes can be identified in cell-free DNA (cfDNA) from patient blood samples using this method (52). The method is therefore useful for molecular monitoring of cancer treatment as well as detecting recurrence and resistance (53). The FDA has recently approved the first “liquid biopsy” test for EGFR mutations in patients with non small cell lung cancer (NSCLC) (53).

## STORAGE OF BIOSPECIMENS

Storage facilities are important factors in maintaining sample quality. The establishment of a storage unit is determined by the type of biobank to be established, the type of samples to be stored, the storage period of the samples, the intended use of the samples, and the financial resources (4). Additionally, the presence of infrastructure facilities such as an electrical power system, backup systems, transport conditions, and on-site support services should be considered. Compared with the modest conditions required for the storage of FFPE blocks (such as room temperature below -25°C, air conditioning, space), a biobank storage unit requires more sophisticated conditions and expensive equipment.

The sample freezing temperature is determined by the amount of water and other tissue components within the sample (4). Biospecimens can therefore freeze over a range of temperatures. All biological specimens contain degradative molecules affected by the temperature. As the ambient storage temperature decreases, the activity of the protein within the biospecimen also decreases. The optimal storage temperature should therefore be below the threshold temperature of protein activity (4,54). Optimal storage temperatures are below -132°C, which is the glass transition temperature (Tg) of pure water. Most of the chemical and physical reactions that cause deterioration of the specimen slow down below this temperature.

It is possible to store specimens at low temperatures using mechanical freezers or liquid nitrogen-based (LN2) cryogenic storage (4,40,54). The LN2-based storage units provide effective and long-term storage and are preferred to mechanical freezers in areas where the power supply is unreliable (4,40). LN2-based storage units are composed of two main groups as the smaller *aluminum dewars* and the *large storage units *(4,40). Both are double-walled, vacuum-insulated storage units that efficiently hold LN2. The units have different sizes and specimen capacities. *Aluminum Dewars* are small and transportable containers that can be installed conveniently in labs and they are readily accessible (4,40). They provide a stable storage temperature and low LN2 usage but most of them require manual filling of the LN2 to maintain the temperature. Dewars typically lack full monitoring and LN2 level control options. *The liquid storage* units are medium to large storage areas that provide long-term storage of specimens (4,40). Due to their size, they may be installed in a lab or require a special area. The majority of these units have auto-fill capabilities for temperature and LN2 level with a monitoring system. Even so, manual verification at regular intervals is important to ensure specimen integrity in these units. Biospecimens can be stored in the liquid or vapor phase of liquid nitrogen (4,40,54). In vapor phase containers, the biospecimens are stored above the liquid phase nitrogen but are surrounded by the vapor (gaseous) phase. Storage in the LN2 vapor phase (≤150°C) provides appropriately low temperatures maintained below the Tg (-132°C) and protects biospecimens from the risk of contamination and safety hazards related to liquid phase storage. Vapor phase LN2 storage is therefore usually preferred to liquid phase LN2 (-196°C). However, one must consider that liquid phase LN2 provides a stable temperature at -196°C. Oxygen level sensors should be used and calibrated when LN2 freezers are used. Protective goggles and gloves must be used. Appropriate training should also be provided as a part of an SOP related to health hazards and safety precautions (4,40).

The container system is another important component of the storage unit. There are three main types of containers: screw-cap vials, bags and cryogenic straws (4,40). Screw-cap vials are made from polypropylene and polystyrene in capacities ranging from 0.2 to 5 mL and are recommended for long-term, low temperature storage (4,40,55). Covering of vials with a membrane is important to reduce contamination in liquid nitrogen (4,56). Cell freezing bags, which are commonly used in blood banking, can also be a container of choice for other types of cells or tissues (4,40). Storage of brain slices within these bags is well-known (57). It is recommended to use vial systems for sample volumes below 5 mL and bags for larger volumes. Cryogenic straws are hermetically sealed and designed for the safe storage of specimens within the liquid phase of nitrogen (4,40). These straws are made from chemically inert and biocompatible material and show physical characteristics resistant to ultra low temperatures and storage pressures (4,40). They are therefore stable at compelling circumstances such as snap-freezing, exposure to low temperatures for a long time (years), or several freeze-thaw cycles. Wrapping frozen tissue slices in aluminum foil also helps to minimize tissue desiccation (55).

When selecting storage containers for biospecimens, the following issues should be considered (5)

- Sample volume 
- Cooling and warming rates required
- Potential risk of contamination of the sample or the environment 
- Storage temperature and conditions 
- Available space for storage 
- Frequency of access 
- Specimen identification requirements (identification labels without personal identifiers that are compatible with the storage temperature and medium, eye-readable codes)
- Specimen preparation and processing techniques 
- Economic situation 

All human specimens should be treated as potential biohazards. Taking precautions against the risk of contamination of laboratory workers who handle the specimens in the laboratory or during transportation is therefore important and in fact a part of good laboratory practice (5).

### Mechanical Freezers

Mechanical freezers are available in varying sizes, configurations, and electrical voltages (4,54). Since these are devices attached to power systems, a back-up power plan and emergency response plan must be established. Lower cost of initial investment and easier access to samples are the main advantages of mechanical freezers (4,54). Storing biospecimens at low temperatures (-20°C) for the short term or at ultra low (-80°C and -150°C) temperatures for the long term is possible but the use of -20°C is gradually decreasing as tissue degradation occurs at this level (4,54). Most of the centers prefer to use ultra low temperatures (-80°C; -150°C), because ice crystals can develop within the biospecimens at temperatures near -70°C (4,54). Cascade compressors might produce temperatures as low as -140°C but require constant electrical power to maintain these temperatures. The temperature stability of freezers is influenced by several factors such as the ambient temperature, humidity, open doors during sample loading, and frost within the freezer. Freezers should therefore be placed in rooms with proper air-conditioning and any frost should be removed regularly (4,54).

### Refrigerators

Refrigerators are used where the durability of the material being stored is preserved by storage below ambient temperature. Storage at 4°C could be an intermediate step before preparation for ultra low temperature storage (4,54). The temperature stability and back-up power plan are important for refrigerators as for mechanical freezers (4,54).

### Ambient Temperature Storage

Formalin-fixed paraffin-embedded tissue specimens can be stored at room temperature in routine pathology lab practice. Recently developed biological storage matrices allow for the long-term maintenance of biological components at room temperature (4,54). These matrices can be helpful in the absence of mechanical or cryogenic equipment due to practical or financial issues and enable the storage of many types of tissue specimens such as formalin-fixed, PAXgene-fixed, ethanol-fixed, paraffin-embedded and lyophilized samples (58). Other studies have suggested different methods to preserve nucleic acids in FFPE tissue blocks. Baeane-Del Valle et al*. *have examined the effects of tissue block age on FFPE tissues from radical prostatectomy specimens from patients with prostate cancer using a number of RNA in situ probes (59). They found a decrease in signals after 5 years and a significant decrease after 1 year. They also showed that storing unstained slides in recent cases (< 1 year old) in the cold (–20°C) preserves RNA in situ hybridization signals, and was superior to leaving the tissues in FFPE blocks stored at room temperature. Therefore, the authors suggested this simple solution (cold storage of unstained slides) to better preserve invaluable RNA-based information currently locked in massive FFPE archives (59).

The effects of various storage temperatures on the molecular integrity of frozen tissues have been studied. Some studies have demonstrated reduced RNA integrity in specimens stored at −70°C or −80°C for 5 years or more (60,61). Whether the reduced RNA integrity was due to the peculiar inherent characteristics of the tissue samples or storage conditions was not clear in these studies. Conversely, the integrity of RNA was assessed with the reverse transcription polymerase chain reaction (RT-PCR) after the storage of brain autopsy tissue for 15 years at −70°C and no RNA deterioration was reported (62). In contrast to the conflicting results for RNA, DNA integrity is usually preserved with long-term storage at -80°C (61). Similarly, the proteome may be preserved for years when tissue if stored at or below -70°C (63). In another study, the activity of epidermal growth factor receptor was investigated in excision materials of patients with breast cancer and there was no difference related to the storage temperatures (liquid nitrogen, -70°C, -20°C) (64). It has been suggested that a minimum temperature of -80°C should be preferred for long-term storage. Although storage at -150°C may reduce the effects of temperature fluctuations resulting from opening freezer doors, additional data are needed before these costly freezers are preferred.

The effects of different temperatures on the storage of blood specimens have also been analysed. Collected blood samples should be processed as soon as possible to maintain biomolecule yield and to prevent degradation (47). It has been demonstrated that DNA could be extracted with admissible yield and quality from blood samples that are stored at room temperature, at 4°C, and at -20°C for a maximum period of 1 month (65). As the storage term becomes prolonged, the erythrocytes and some of the leukocytes will undergo lysis and consequent loss in the amount of extracted DNA (65). In such circumstances, freezing the blood samples at -80°C has been recommended to avoid lysis and to improve the DNA yield (66). Extracted DNA from blood samples can maintain its stability at 4°C for weeks, at -20°C for months, and at -80°C for years. However, RNA lability and degradation begins at temperatures higher than -80°C (67). Interestingly, miRNA, a species of RNA, can maintain its integrity without prominent degradation for years in plasma samples that are stored at -80°C (68).

### Freeze-Thaw Cycles****

Freeze-thaw cycles can be damaging to the biomolecules and cells intended for analysis. Repeated cycles lead to increased cell death via apoptosis and necrosis (4). Therefore, it is important to aliquot the biospecimens to the proper size in order to minimize the number of these freeze-thaw cycles before they are used. If aliquoting is not possible, placement of samples on dry or wet ice during sampling will help to maintain the vitality of the biospecimens (4,54). As far as we know, preservation of the sample integrity is possible below the level of Tg (-132°C) where biochemical activity of the cell is almost stopped (4,54). A specimen therefore experiences a micro-thaw event each time it is warmed above Tg. There are several factors that can cause temperature fluctuations such as power outages, mechanical failure of the freezer, or frequent door openings (4,54).

Most of the studies investigating the freeze-thaw cycles address the integrity of RNA since it is the most sensitive biomolecule of unfixed tissue. Some of the studies have shown that repeated freeze-thaw cycles (as few as 2 thaw events) are sufficient to reduce RNA quality, particularly in autopsy brain tissue (69,70). However, other studies have not found any alterations in RNA quality, gene expression profile, or protein expression in frozen ovarian and brain tissue samples (71,72). Additionally, it has been reported that at least 3 freeze-thaw cycles can be performed without loss of RNA quality in ovarian tissue samples (72).

Similarly, minimal RNA degradation was reported after 6 freeze-thaw cycles in tonsillar tissue in another study emphasizing the importance of short-term (i.e., 5 minute) thaw cycles for RNA integrity (73). The reason for the contradictory RNA integrity results reported from various studies could be the lability of biomolecules in different tissue types, the varying nuclease content, and the thawing conditions. In fact, total thaw time at ambient temperature has been considered to have a stronger effect on RNA integrity than the number of freeze-thaw cycles (73,74). However, a total thaw time of less than 30 minutes at ambient temperature has been reported to suitable for the preservation of RNA integrity, regardless of the number of freeze-thaw cycles (73,74). In addition, aliquots stored in RNAlater (Qiagen, Valencia, CA) have been reported to have a higher quality RNA than snap-frozen samples and also to be more resistant to freeze-thawing (69). Preserving specimens in RNAlater mitigates the effect of thawing on RNA integrity.

Freeze thaw cycles also influence DNA, RNA, and protein stability in blood samples. The number of freeze-thaw cycles of blood samples should be minimized as in frozen tissue samples. Aliquoting as well as pre-extraction of stable molecules (DNA) can be used for this purpose.

Finally, extra care is required regarding the aliquoting and documentation of the biospecimens. Biospecimens collected from the same patient should be aliquoted and then preserved in two different repositories so as to protect them from the adverse effects of undesirable conditions such as power outage. Conditions for preventive interventions in case of failure should be provided. For example, mechanical freezers should be protected from power outages particularly via an electrical backup system (2 uninterruptible power supplies). The location of the biospecimens within the storage, the information regarding the amount of tissue used for each assay, and the residual tissue samples should also be recorded carefully.

## QUALITY CONTROL PROGRAM IN BIOBANKS

Biobanks are very important centers for understanding the mechanisms of diseases, developing clinical biomarkers associated with diseases, and discovery of new drugs. These repositories where the properties of the relevant material are defined and preserved in high quality therefore require the implementation of quality control and quality assurance plans at each stage (Table 1) (75).

**Table 1:** Some of the terms related to Quality and their explanations.

**Quality management system (QMS)**	It defines the quality policy and objectives of the biobanks and ensures the successful implementation of quality assurance and quality control
**Quality control (QC)**	It covers daily scientific and technical tasks carried out by laboratory staff under the supervision of the responsible physician (e.g., the pathologist)
**Quality assurance (QA)**	It is a component of quality control. In addition, it covers the responsibility of “applying medical provision”. It includes the processes of achieving excellent medical results in biobanking services
**Certification**	It is a third party to issue written assurance (certification) that a product, process, or service during biobanking meets certain requirements.
**Accreditation**	It is a recognition of the competence of the biobank in performing the relevant tasks by the procedure officially implemented by an independent competent authority. Accreditation requires method verification.

Biobanks are resources that work as a library within the medical sciences. It is therefore very important to record and ensure the safety of biobank material data. During biobanking, data processing and sample processing work packages as well as quality control mechanisms should be established. The aim of data processing is to record the demographic and clinical data of the patients and the identity information on the sample using a unique and holistic approach (1). The use of standardized methods during registration is important to ensure quality. For example, the use of the BRISQ (Biospecimen Reporting for Improved Study Quality) reporting system allows for better storage, recording and comparison of the data records (Table 2) (76–78).

**Table 2:** Items included in the BRISQ (Biospecimen Reporting for Improved Study Quality) reporting system.

Type of biospecimen
Anatomical region
Patient’s history of disease
Clinical features of the case
Survival status
Clinical diagnosis
Pathologic diagnosis
Type of material
Storage method of the material after its receival
How the material is protected in the biobank
Characteristic features of the contents of the preservative
Storage temperature
Duration of storage
Temperature during delivery of the material to the relevant section
Percentage of areas such as tumor necrosis as a result of material evaluation

Quality management and standardization in biobanks is ensured by the necessary guidelines and standard operating procedures (SOPs). There are standard operating procedures for biobanking published by the International Society for Biological and Environmental Repositories (ISBER) and the National Cancer Institute Biorepository and Biospecimen Research Branch of the National Cancer Institute (NCI BBRB). These procedures, which include guidance on issues such as material collection, processing, and use in addition to education and ethics can be used by bringing them into conformity with the legal framework of the biobank and the country’s legislation (79). Operability of national or international networks that have been already established or are likely to be established for similar purposes, usually in close geographic regions, should be ensured. The Molecular Pathology Working Group of the European Society of Pathology (ESP) works in collaboration with other European organizations (such as the Organization of European Cancer Institutes, and the European Biobanking Infrastructure), and continue their research on pathology-centered biobanks (2).

The ISO 9001, ISO 17025 and ISO 15189 standards published by the International Organization for Standardization (ISO) are not specific to biobanking but have been previously in use for this purpose (80). Standards specific to biobanking (ISO/TC276) were determined with the publication in 2018 of ISO 20387 that includes the requirements for biobanking. ISO 20387 includes standards for the collection, recording, cataloging, classification, processing, reproduction, packaging, storage, disposal, distribution, transportation of materials, as well as security measures, risk management and personnel-related issues. The Accreditation Institute of Turkey, a member of the Accreditation working group of the European Union Accreditation Agency, has taken the ISO 20387 biobanking standard into consideration. Studies for its validation in European Countries including Turkey are still continuing (81,82).

Within the framework of these standards, quality assurance programs (QAPs) regarding educational and infrastructure issues in diagnostic research should be established in biobanks. Improvement of the quality levels in the pathology-centered biobanks in accordance with the accreditation programs of CAP (College of American Pathologists) has been planned.

Quality assurance and quality control programs should be defined in detail for the biobanks that make up a large number of important organizations established to facilitate diagnosis and research. The quality assurance program aims to minimize the impact of preanalytical variables on the biospecimens stored in biobanks. Standard operational procedures (SOPs) which are understandable, clear and easily monitored by the employees should be developed and implemented. Quality assurance programs should also be applied to determine the effect of the SOPs and preanalytical variables on biospecimens stored in the biobank (83,84).

### Quality Assurance Program in Biobanks

The components of the quality assurance program in pathology-centered biobanks include technical and operational elements, the role of the pathologist and the employees, and the quality of the banked sample (Table 3 Table 4). Since the quality assurance program in biobanks cannot obtain data without a written record, everything related to quality should be recorded. A quality assurance program and a quality assurance committee should be established for each biobank. Committee members may consist of a biobank manager, quality officer, laboratory officer pathologist, and a technician. In addition, employment of personnel trained in the field of quality control may be considered within the scope of possibilities (83,85,86). 

**Table 3:** Main elements of the Quality Assurance program.

**Technical and operational elements**	Identification, registration and acceptance of the material, Processing of the material Procedural guidelines Device maintenance Storage of the material
**The role of the pathologist**	Evaluation of tumor ratio in materials Correct separation of tissues with tumor and without tumor (control tissue) Conferences, seminars and training programs
**Quality of the materials stored in biobanks**	Quality of DNA, RNA and protein, adequacy of their quantity and purity Adequacy of the registration data of the material

**Table 4:** Important issues for Quality Control and Quality Assurance.

Establishment of a quality assurance program and sharing of responsibilities
Preparation of standard operating procedures
Device instruction manuals, maintenance, calibration, repair and registration of devices and equipment
Provision of infrastructure, workspaces and equipments
Control and recording of the physical and chemical properties of the environment
Quality, quantification and purity tests for DNA, RNA and proteins to determine quality of the material
Microorganism contamination tests and cross-contamination tests between biospecimens
Establishing and securing the safety of appropriate data processing technology systems
Taking measures for biological, physical, and chemical security
Establishment of a waste disposal system
Human resources and staff training programs
Consideration of ethical and legal regulations
Certification and Accreditation
Establishment of networks, communities, and working groups between biobanks
Seminars and congresses

### The Role of the Pathologist in Quality Processes

The pathologist is responsible for managing and regulating a quality control program in biobanks. In addition to macroscopic evaluation and identification of biospecimens stored in the biobank, the role of the pathologist in the differentiation of the lesion (tumor) tissue from control tissue is very important (87). Histomorphological evaluation during the quality control of the tissue specimens is necessary for the confirmation of the pathological diagnosis and determination of the disease status and tumor-necrosis-stroma-inflammation-normal tissue ratios. In addition, the pathologist is responsible for detecting tumor specimens with heterogeneous cell populations and performing manual macrodissection or manual/laser microdissection if necessary. Tissue microarray (TMA) preparation can facilitate the diagnostic procedures, conduct of research, and the financial processes in some cases (83).

Reevaluation of stored tissue specimens by a pathologist using Hematoxylin & Eosin-stained slides, and performing comparisons between frozen and FFPE tissue specimens can help to evaluate the quality of the stored material (88). In addition, pathologists are responsible for the quality control of molecular pathology procedures related to the tumor biobank.

## FINANCING BIOBANKS

Calculating the cost and budget of a biobank requires a detailed plan to be prepared for the installation, operation, development and long-term sustainability of the relevant biobank. Large material resources are therefore needed (1,5,89,90). To this end, public institutions, private enterprises or associations can contribute to this financing. Consequently, financing may be provided by a public model or the private financing model in general. Funds for a pathology-centered biobank in our country can be provided by the public, various state institutions (e.g., TUBITAK) or private universities.

**Figure 1:**
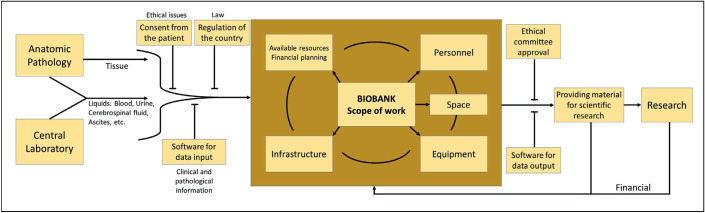
The organizational scheme of a biobank.

Biobank expenditures are analyzed in two phases as initial and operational (Table 5). The initial stage involves the planning and establishment of the biobank. The first budget prepared should cover the determination of and the necessary arrangements to be made for the biobank area, establishment of an infrastructure suitable for the workflow, the provision of the necessary devices and equipment, establishment of appropriate recording and monitoring software/hardware information systems, identification and provision of preventive measures against natural disasters and occupational risks, planning and monitoring tissue safety, and the provision of ventilation and proper air conditioning. The size and scale of the biobank, the size of the area required, and the quality of the equipment and services are the factors that determine the amount of initial funding (89). At the operational stage, personnel costs (such as the biobank manager, pathologist, technician, data assistant, quality control personnel) are expected to take up most of the budget. The cost of consumable materials should also be calculated, in addition to utilities, electricity, water, natural gas, and medical waste disposal. Consultancy services related to quality control and assurance, and ethical or legal matters should also be included in the financial plan. In addition, the financing of educational meetings such as training programs, seminars and congresses as well as certification and accreditation costs should be included in the budget so that biobanks can be established and developed and high quality services then purchased (89). The organizational scheme of a biobank is summarized in Figure 1.

**Table 5:** Some expenses related to the establishment and operation of the biobank.

Biobank area
Devices and equipment
Infrastructure
Personnel expenses
Consumables
General expenses such as electricity, water, natural gas
Maintenance of devices, technical support
Ethical and legal consultation support
Training
Transportation of materials
Certification and accreditation

The cost-effectiveness of the biobank process should be considered carefully. Weighing the sustainability costs of each project in terms of duration and project outcome is important, and costs should be delineated. Even if a biobank’s ability to recover costs is often limited, it can generate income from the research budget, the donations, and the user fees it will receive from the patients. After a certain period of time, biobanks may become financially independent.

Consequently, a pathology-centered biobank offers sig-nificant advantages (Table 6). First of all, it ensures correct use of tissue specimens in the diagnosis of diseases and in the definition of targeted individualized therapies. Effective selection of the necessary tissue specimens for research projects can also be realized within the same process. Thus, each of the tissue-related processes is realized in the presence of experienced personnel and they can all be gathered at a single center. This provides effective and significant savings at both the tissue and cost levels (9,10).

**Table 6:** Advantages of Pathology-centered biobanks.

Rapid and effective use of tissue specimens for diagnostic and therapeutic purposes
Availability of personnel experienced in recording, processing and storage of tissue specimens
Reducing costs by gathering materials in one center
Reduction of costs of sampling for use in research studies

## Conflict of Interest

The authors declare no conflict of interest.
